# A Novel Immunodiagnostic Assay to Detect Serum Antibody Response against Selected Soluble Egg Antigen Fractions from *Schistosoma japonicum*

**DOI:** 10.1371/journal.pone.0044032

**Published:** 2012-08-31

**Authors:** Yinchang Zhu, Wanquan Hua, Ming Xu, Wei He, Xiaoting Wang, Yang Dai, Song Zhao, Jianxia Tang, Shixia Wang, Shan Lu

**Affiliations:** 1 Key Laboratory on Technology for Parasitic Diseases Prevention and Control, Ministry of Health and Jiangsu Provincial Key Laboratory on Molecular Biology of Parasites, Jiangsu Institute of Parasitic Diseases, Wuxi, Jiangsu, People's Republic of China; 2 Wuxi Hospital for Maternal and Child Health Care, Wuxi, Jiangsu, People's Republic of China; 3 China-US Vaccine Research Center, Jiangsu Province Hospital, Nanjing, People's Republic of China; 4 Laboratory of Nucleic Acid Vaccines, Department of Medicine, University of Massachusetts Medical School, Worcester, Massachusetts, United States of America; Queensland Institute of Medical Research, Australia

## Abstract

**Background:**

Schistosomiasis japonica remains a real threat to public health in China. The currently used immunodiagnostic assays are sensitive and have a certain degree of specificity, however, they all use complex crude antigens, are based on detection of schistosome-specific antibodies, and have been shown to cross-react with other parasitic diseases. Therefore, these assays cannot be used to evaluate chemotherapy efficacy. The development of highly sensitive and highly specific immunodiagnostic techniques that can monitor the decline of antibodies specific for *S. japonica* will be extremely valuable as part of the ongoing strategy to control schistosomiasis in endemic areas. Here we report on the identification of unique fraction antigens of soluble egg antigen (SEA) to which the antibodies disappear 7 weeks after effective treatment. Furthermore, we use these SEA fractions to develop a modified assay with both high sensitivity and specificity.

**Methodology/Principal Findings:**

SEA of *S. japonicum* was fractionated by electrophoresis using 7.5% SDS-PAGE under non-reducing conditions. The SEA fraction antigens to which antibodies were decreased soon after treatment were collected and used as the detection antigens to establish the FA-ELISA. Sera from patients with acute and chronic schistosomiasis infection, healthy people, and those with other parasitic diseases, were used to evaluate their sensitivity and specificity. Furthermore, sera from patients with chronic schistosomiasis infection were evaluated before and after treatment at different time points to evaluate their chemotherapeutic efficacy.

**Conclusion/Significance:**

We demonstrated that this novel FA-ELISA provided high sensitivity and specificity, with very low cross-reactivity, and can serve as an effective tool to determine the efficacy of chemotherapy against *S. japonicum*.

## Introduction

Schistosomiasis is caused by adult blood flukes that deposit eggs into blood vessels around the liver, gut, or bladder of the infected host. Six species of schistosomes are known to infect humans and cause schistosomiasis. Approximately 779 million people are at risk of being infected with the disease in more than 70 countries throughout the world [1]. Schistosomiasis japonica, caused by *Schistosoma japonicum*, is a serious disease that is endemic in China and, to a lesser extent, the Philippines. Despite over 60 years of efforts, schistosomiasis japonica remains a real threat to public health in China. Approximately 240 million people are at risk of infection with the disease and 413,000 people are actively infected, particularly in the five provinces in the lake and marsh regions (Hubei, Hunan, Jiangxi, Anhui, and Jiangsu) and two inland provinces with large mountain ranges (Sichuan and Yunnan) [2]. At the same time, the prevalence rate of schistosomiasis and the intensity of infection have declined in China [3]. The use of immunodiagnostic assays has contributed to such progress but also points to the need for the development of improved assays, especially for the purpose of monitoring therapeutic effects in infected populations.

Immunodiagnostic techniques provide several unique advantages. They are usually sensitive, easy to perform, and function as excellent epidemiological tools for the screening of targeted populations in schistosome-endemic areas [4], [5], [6]. A number of immunodiagnostic techniques are currently used, including enzyme-linked immunosorbent assay (ELISA) [7], dye dipstick immunoassay (DDIA) [8], the indirect hemagglutination assay (IHA) [7], the circumoval precipitin test (COPT) [9]. These assays all use complex crude antigens, such as soluble egg antigen (SEA), and are based on the detection of schistosome-specific antibodies in infected hosts. Although these assays are sensitive and have a certain degree of specificity, they have been shown to cross-react (up to 64–84% in some cases) with other parasitic diseases, such as paragonimiasis, clonorchiasis, and fasciolopsiasis [10], [11]. Furthermore, these immunodiagnostic assays cannot be used for the evaluation of chemotherapy efficacy, which needs monitoring in order to determine potential decreases of schistosomiasis japonica in infected hosts given that the current assays for detecting circulating schistosome antigens are not much more sensitive than the direct examination of infection by microscopy [12], [13], [14]. Therefore, the development of highly sensitive and highly specific immunodiagnostic techniques that can monitor the decline of antibodies specific for schistosomiasis japonica will be extremely valuable as they can be used to evaluate chemotherapy efficacy as part of the ongoing strategy to control schistosomiasis in endemic areas. In the present study, we report the identification of unique fraction antigens of SEA to which the antibodies disappear soon after effective treatment. We also report on the use of these SEA fractions to develop a modified ELISA with both high sensitivity and specificity.

**Figure 1:**
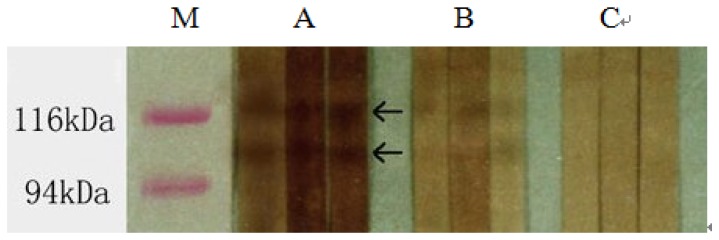
Western blot analysis of SEA fractions recognized by sera from three rabbits before and after treatment at different time points. (A). SEA fractions recognized by the sera from infected rabbits before treatment; (B). SEA fractions recognized by the rabbit sera at 7 weeks after treatment; (C). SEA fractions recognized by the rabbit sera at 11 weeks after treatment.

## Materials and Methods

### Ethics statement

Human studies were approved by the Institutional Review Board (IRB00004221) of Jiangsu Institute of Parasitic Diseases, Wuxi, China. For the human studies, questionnaire surveys, physical examinations, and laboratory assays were conducted following informed consent procedures. Patients were given the right to withdraw from the study at any time, without consequence. Written informed consent was obtained from all adult participants (including patients and healthy controls) or from the parents or legal guardians of minors. The animal protocol was approved by the Jiangsu Institutional Animal Care and Use Committee (IACUC), according to the administration of lab animals issued by the Ministry and Technology (Beijing, China).

**Table 1:** Testing of serum samples from patients infected with schistosome or other parasites and healthy donors by FA-ELISA, SEA-ELISA, and S-ELISA.

Subjects	No. of serum samples	No. (%) of positive detection by different assays		
		FA-ELISA	SEA-ELISA	S-ELISA
*Schistosomiasis* *				
Acute	67	61 (91.0%)	63 (94.0%)	57 (85.1%)
Chronic	94	90 (95.7%)	91 (96.8%)	83 (88.3%)
*Other parasites*				
Clonorchiasis	40	0	2(5.0%)	1(2.5%)
Fasciolopsiasis	27	0	0	0
Paragonimiasis	10	0	3 (30%)	1 (10%)
Healthy donors	60	0	1 (1.7%)	1 (1.7%)

* Cases diagnosed by stool examination (100% positive).

### Preparation of SEA

Soluble egg antigen (SEA) from *S. japonicum* was made with purified eggs from the liver of infected rabbits, as previously described [10]. Briefly, eggs of *S. japonicum* were collected from infected rabbit livers and separated from the host tissue. Eggs were then transferred in a 0.9% NaCl solution and homogenized for 1 hour on ice. The supernatant was collected as SEA after centrifugation at 20,000 rpm at 4°C for 1 hour.

**Table 2:** Comparison between FA-DIGFA and SEA-DIGFA for testing sera from patients infected with schistosome and other parasites.

Subjects	No. of serum samples	No. (%) of positive detection by different assays	
		FA-DIGFA	SEA-DIGFA
Chronic schistosomiasis*	54	51(94.4%)	52(96.3%)
Clonorchiasis	30	0	3(10%)
Paragonimiasis	19	0	5(26.3%)
Healthy donors	50	0	0

* Cases diagnosed by stool examination (100% positive).

### Animal studies

Seven rabbits were each infected with 500 cercariae of *Schistosoma japonicum*. At 45 days post-infection, the feces of the infected rabbits were collected and examined under the microscope. *S. japonicum* eggs could be detected in the feces of all seven of the infected rabbits. The infected rabbits were immediately treated orally with praziquantel (150 mg/kg) and again one week later.

Serum was collected from the ear vein before and after infection at different time points until week 24. Infected rabbits were sacrificed 24 weeks after treatment in order to confirm treatment effects (i.e., the absence of adult worms in the mesenteric vein).

**Table 3:** Monitoring the disappearance of schistosome specific antibodies using FA-ELISA and SEA-ELISA.

Chemotherapy	No. of subjects	No. (%) of positive detection by different assays	
		FA-ELISA	SEA-ELISA
*Before treatment* *	30	28 (93.33%)	29 (96.67%)
*After treatment*			
8 months	22	14 (63.64%)	20 (90.91%)
12 months**	23	10 (43.48%)	19 (82.61%)
18 months	22	2 (9.09%)	13 (59.09%)

* Cases diagnosed by stool examination (100% positive).

** Stool examination 100% negative by Month12 following treatment.

### Preparation of SEA fractions

SEA of *S. japonicum* was fractionated by electrophoresis using 7.5% sodium dodecy1 sulfate-polyacrylamide gel (SDS-PAGE) under non-reducing conditions. The molecular weights (MW) of fractionated bands were estimated based on the standard MW marker. The fractionated antigens were then transferred to the nitrocellulose membrane and probed with sera collected from rabbits included in the study at Weeks 0 (prior infection), 5, 7, 9, 11, 13, 16, 20, and 24 post treatment. The sera were diluted to 1∶400. Staphylococcal protein A conjugated horseradish peroxidase (HRP) (Shanghai Bio-products Com.) was used to detect the antibody. The region containing the special SEA fractions was cut from SDS-PAGE gel and the SEA fractions were eluted by using the Bio-Rad Electro-Elutor cell. Eluted fractions were collected, concentrated, and stored at −60°C until use.

**Table 4:** Comparison of 16 different diagnostic systems for schistosomiasis japonica.

Diagnostic Methods*	% of positive schistosomiasis parasite antigen detections				
	Healthy donors	Chronic schistosomiasis patients	Paragonimiasis patients	Clonorchiasis patients	Score
ELISA1	0	60	40	35	76.5
ELISA2	20	62	65	35	66.7
ELISA3	0	32	10	5	-
ELISA4	0	60	25	25	79
**ELISA5**	**0**	**90**	**0**	**10**	**92.5**
ELISA6	0	92	85	0	86.7
ELISA7	0	90	30	0	90.5
ELISA8	3.3	92	95	15	83
IHA1	0	92	55	0	89.7
IHA2	3.3	94	75	10	85.2
IHA3	0	90	40	5	89
IHA4	3.3	92	60	0	88
IHA5	3.3	52	30	5	-
IHA6	6.7	36	20	0	71.3
LA	3.3	78	80	0	81.1
DIGFA	20	86	45	25	80.1

* **ELISA1, 2, 3** for detection of schistosome antigens with monoclonal antibodies; **ELISA 4, 5, 6, 7, 8** for detection of anti-schistosome antibodies with crude schistosome antigens except **ELISA 5** which used the 107–121 kDa fraction antigens; **IHA** for detection of anti-schistosome antibodies with crude schistosome antigens; **LA** (latex agglutination) for detection of anti-schistosome antibodies with crude schistosome antigens **DIGFA** for detection of anti-schistosome antibodies with crude schistosome antigens.

**ELISA 3** and **IHA 5** were not scored due to certain technical errors during the test.

### ELISA assay

#### Fraction antigen ELISA assay (FA-ELISA)

SEA fraction antigens at 5 µg/ml were coated on the ELISA plate. The plate was blocked with 1% BSA in PBS for 30 min. The diluted human sera at 1∶200 were added, incubated at 37°C, and washed with 1% Tween in PBS. HRP-protein A (1∶40) (for rabbit sera test) or HRP-anti-human IgG (for human sera test) was added and incubated at 37°C. Then the substrate 3,3′,5,5′-tetramethy1 bonzidine (TMB) was added and 50 µl of 2N H_2_SO_4_ was used to stop the reaction. The results were read using an ELISA plate reader at 450 nm. Positive and negative controls were included on each plate.

#### SEA- ELISA

In this assay, 10 µg/ml of SEA was coated on the plate for detecting antibody responses. Anti-human IgG conjugated with HRP was used as the secondary antibody. The same methods as those used for the FA-ELISA (2.4.1) were used from this point forward.

#### Double antibody sandwich ELISA (S-ELISA)

Circulating antigens in patients infected with schistosomiasis japonica were tested by S-ELISA. In this assay, 200 µg/ml of rabbit immunoglobulin was extracted from rabbit sera infected with 1500 cercariae of *S. japonicum* and was coated on the plate as the capture antibody. Monoclonal antibody, NP28-5B conjugated with HRP (a gift from Dr Xiaohong Guan, Nanjing Medical University), was used as the detecting antibody.

### Dot immunogold filtration assay (DIGFA)

#### Fraction antigen DIGFA (FA-DIGFA)

SEA fraction antigen (1 µg/µl) and the control human IgG (1 µg/µl) were dotted on the filter membrane (0.45 µm, Millipore, HAWP02500) then blocked three times with 1% BSA before adding 20 µl of testing serum sample on the dotted paper. After washing with 0.01% PBST phosphate buffered saline containing 0.01% Tween-20 (PBSTX), 100 µl of diluted anti-human IgG conjugated with colloidal gold (Lifeholder, G11418) was placed on the filter membrane as the detecting antibody. Then 100 µl of pH 7.4 PBS was added and the results were read 2–5 min later. If both dots were red, the sample was positive; if only the control dot was colored, the sample was considered negative.

#### SEA-DIGFA

SEA (1 µg/µl) and the control human IgG (1 µg/µl) were dotted on the filter membrane and the remaining procedure was the same as described for FA-DIGFA (2.6.1).

### Sera

#### Sera from patients with acute S. japonica

Sera were collected from patients with acute schistosomiasis who had contact with infected water, had a positive egg test, as determined by stool hatching test, and had typical clinical manifestations from Jiangxi (N = 34) and Anhui endemic areas (N = 33).

#### Sera from patients with chronic S. japonica

Sera were collected from 94 patients (for the ELISA experiment) and 54 patients (for the DIGFA experiment) from schistosomiasis japonica endemic areas who had confirmed schistomiasis, as determined by stool examination.

#### Healthy control sera

60 sera samples (for ELISA) and 50 samples (for DIGFA) were collected from healthy individuals from non-endemic areas.

#### Sera from patients with Clonorchiasis

Serum was collected from 47 patients (for ELISA) and 30 patients (for DIGFA) from endemic areas who were infected with *Clonorchis sinensis* as verified by stool examination.

#### Sera from patients with Fasciolopsiasis

Serum was collected from 27 patients from an endemic area who were infected with *Fasciolopsis buski* as verified by stool examination.

#### Sera from patients with Paragonimiasis

Serum was collected from 10 patients (for ELISA) and 19 patients (for DIGFA) from endemic areas who were infected with *Paragonimus westermani*. These patients had typical clinical manifestations and a positive immunodiagnostic test for paragonimiasis (a gift from Prof. Zhang, Nanjing Medical University, China).

### Evaluation of chemotherapy efficacy

#### Patient selection

Thirty patients with chronic schistosomiasis japonica were selected from a transmission controlled endemic area in Jiangsu, China. Infection in these patients was verified by stool examination then treated with praziquantel (60 mg/kg) for two days. One year later, stool examination was performed again to confirm that these patients were free from any remaining infection.

#### Sera

Sera from these 30 patients were taken before treatment and at 8, 12, and 18 months after treatment. Sera were stored in −60°C until use.

## Results

### Analysis of SEA fraction antigens from *S. japonicum*

Based on molecular weight, SEA was separated into 23–28 different fractions by 7.5% SDS-PAGE. Western blot analysis showed that 18 bands were recognized by infected rabbits' sera before treatment. Their molecular weights were around 220, 193, 177, 168, 165, 142–157, 132, 121, 107, 78, 73, 69, 55, 53, 79, 37, 30 and 28 kDa respectively. The reaction of the antibodies to 107 kD and 121 kDa fractions was much weaker when sera from rabbits at 7 weeks after treatment were used. Reactions to 107 kD and 121 kDa fractions disappeared when sera from rabbits at 11 weeks after treatment were used, while reactions to other fractions could still be detected using the same rabbit sera (Fig. 1).

The 107–121 kDa fraction bands were cut from SDS-PAGE gel and eluted in an electro-elutor. The concentrated 107–121 kDa fraction antigens from SEA were recovered by 7.5% SDS-PAGE and used as the coating antigen in the subsequent ELISA assay. An average of 1.1 mg 107–121 kDa antigen fractions was prepared from 17 mg of SEA (at a ratio of 66µg fraction antigen/1 mg of SEA).

### Sensitivity and specificity of FA-ELISA assay

Sera from 27 healthy individuals were tested using FA-ELISA. The average OD was 0.117 (SD = 0.057). By using a cut-off value as the average OD plus three times the SD (X +3SD), we assumed the OD value for a positive FA-ELISA result to be ≥0.27. With this assay, sera from patients with chronic schistosomiasis japonica were tested and the positive rate was 93.33% (28/30), whereas the sera from 60 healthy controls were all negative. In the 27 individuals with faciolopsiasis and 40 with clonorchiasis, no positive results could be detected.

### Evaluation of FA-ELISA for sensitivity and specificity

Sixty-seven patients with acute schistosomiasis, 94 patients with chronic schistosomiasis, 27 patients with fasciolopsiasis, 40 patients with clonorchiasis, 10 patients with paragonimiasis, and 60 healthy controls were tested by FA-ELISA, SEA-ELISA, and S-ELISA. The results are shown in Table 1.

### Sensitivity and specificity of FA-DIGFA

54 chronic schistosomiasis patients, 50 healthy controls, 30 patients with *Clonorchis sinensi* infection, 19 patients with *Paragonimus westermani*, infection were tested by FA-DIGFA and SEA-DIGFA assays. The results are shown in Table 2.

### Evaluation of chemotherapeutic efficacy of FA-ELISA

Sera from 30 chronic schistosomiasis patients from a transmission controlled area were followed by FA-ELISA either before treatment or at 8, 12, and 18 months after treatment. The results were compared to those obtained via SEA-ELISA (Table 3).

There was no significant difference on the positive rates before treatment between FA-ELISA and SEA-ELISA. However, at 8 months after treatment, there was a significant difference between the two assays (P<0.05). At 12 and 18 months after treatment, there were also significant differences (both P<0.01). It showed that when using the FA-ELISA, more than 90% of patients among those treated with praziquantel tested negative at 18 months after treatment while using the SEA-ELISA assay was around 40%.

### Results from a national study using the FA-ELISA assay

During the development of the FA-ELISA diagnosis kit, it was evaluated in a double-blind laboratory study organized by the National Office of Endemic Diseases Control, Ministry of Health (MOH), Wuxi, China in June, 1998. The FA-ELISA assay (named ELISA5 in Table 4) received the highest score among 19 different diagnosis kits provided by different labs in China (Table 4).

## Discussion

Monitoring the treatment outcome of chemotherapy is an important component of the strategy to control schistosomiasis. Reliable laboratory tests are urgently needed to monitor the treatment status in infected patients. Most immunologically based diagnostic approaches currently used in endemic regions were developed to detect specific antibodies in patients' sera against crude parasite antigens that are not very effective in serving this purpose. [7], [8], [9] Similarly, recent studies using monoclonal antibodies to detect circulating antigens could not demonstrate high specificity and sensitivity to determine the course of infection in chronic cases of schistosomiasis [12], [13]. The efficacy of such monoclonal antibody-based approaches was not better than the traditional antibody-based detection approaches in evaluating the effect of chemotherapy. In the current study, we attempted to develop a novel and more effective immunodiagnostic assay to address this need.

Our results showed that the FA-ELISA assay had high sensitivity and high specificity, with minimal cross reactivity with other parasite antigens. The positive rate for chronic schistosomiasis detection with the assay was more than 90%, which was not significantly different from the results obtained using the SEA-ELISA. However, results obtained using the S-ELISA assay (detecting circulating antigen) were lower than the other two assays. The specificity of the FA-ELISA was 100% as it does not score positive among healthy individuals and had no cross reactivity against paragonimiasis, fasciolopsiasis, or clonorchiasis. The old SEA-ELISA and S-ELISA showed different levels of cross reactivity, especially with paragonimiasis. The FA-DIGFA also had high sensitivity and specificity as the positive rate for chronic schistosomiasis detection was 94.4% and results were all negative for healthy individuals and in patients with clonorchiasis or paragonimiasis. However, with the SEA-DIGFA, the cross reaction rates with clonorchiasis and paragonimiasis were 10% (10/30) and 26.3% (5/19). Our result indicate that either the FA-ELISA or FA-DIGFA could be applied for diagnosis of schistosomiasis in endemic areas.

The animal experiment results showed that the antibodies to the 107–121 kDa fraction antigens of SEA disappeared earlier in the cured host compared to other fractions. Our assay, using fractions 107–121 KDa, is therefore, proven to be a good candidate for monitoring the efficacy of chemotherapy.

The nature of 107–121 kD fractions is still unknown but it is important in future studies to identify the amino acid sequences of these fractions so that the genes coding for these fractions can be cloned and recombinant proteins similar to these fractions can be produced in higher level and more standardized conditions.

The sensitivities of FA-ELISA and FA-DIGFA were somewhat lower than SEA-ELISA and SEA-DIGFA (Table 1, 2); however, no significant difference was detected when the tests were repeated twice (p>0.05). Because we did not test sera at different levels of infection or at different times post infection, we do not know the results for early infection or low level infection.

At the same time, the reported test in the current study can be used for monitoring of therapeutic effects or for the detection of new infection. This novel test can potentially play a major role in following people who have been treated effectively, especially in China. Currently with the adoption of various strategies in prevention and control of schistosomiasis, among 453 endemic regions, 269 (59.38%) have met the criteria of transmission interruption and another 104 (22.96%) met the criteria of transmission control [15]. In order to achieve full control in China, a comprehensive control strategy with emphasis on infectious source control has been proposed [16], [17]. With this strategy, it is necessary to quickly diagnose new cases or re-infections before effective treatment can be provided. However, due to low infection rates, the common diagnostic methods, such as the Kato-Katz method, have low sensitivity and may miss up to 40–60% of infected cases [18], [19]. At the same time, the existing immunodiagnostic approaches using conventional ELISA or IFA showed low antibody specificity as such antibodies can persist for 3–5 years or even longer after effective treatment [9], [11] and therefore, cannot be used to detect current infection. Using such methods to diagnose infection will lead to overuse of chemotherapy causing waste of resources and induction of drug resistance. Because our FA-ELISA can reliably detect current infection, and have very low cross-reactivity with other parasites, it is an effective approach to make a correct diagnosis and effectively control the source of infection. This will benefit the ultimate control of schistosomiasis throughout China.

It is possible that our technique can be adapted to other schistosome species but we have not yet conducted such study. It will be important to determine whether the antigen used in our study can also be applied to other schistosome species and whether the homologous antigen can be identified with the other schistosome species. These efforts will be facilitated by determining the protein sequence (or at least the partial sequence) of the antigen used in our assay, which may guide the search of homologous antigens for other schistosome species.
